# Phosphatemia is an Independent Prognostic Factor in Amyotrophic Lateral Sclerosis

**DOI:** 10.1002/ana.27252

**Published:** 2025-04-26

**Authors:** Rosario Vasta, Emanuele Koumantakis, Antonio Canosa, Umberto Manera, Maurizio Grassano, Francesca Palumbo, Sara Cabras, Enrico Matteoni, Francesca Di Pede, Filippo De Mattei, Filippo Vergnano, Jessica Mandrioli, Cecilia Simonini, Ilaria Martinelli, Fabiola De Marchi, Letizia Mazzini, Cristina Moglia, Andrea Calvo, Adriano Chiò

**Affiliations:** ^1^ ALS Center, Department of Neuroscience “Rita Levi Montalcini” University of Turin Turin Italy; ^2^ Department of Public Health and Pediatrics University of Turin Turin Italy; ^3^ Department of Clinical and Biological Sciences University of Turin Turin Italy; ^4^ Neurology 1, AOU Città Della Salute e Della Scienza di Torino Turin Italy; ^5^ National Research Council Institute of Cognitive Science and Technologies Rome Italy; ^6^ School of Advanced Studies, Center for Neuroscience University of Camerino Camerino Italy; ^7^ Department of Biomedical, Metabolic and Neural Sciences University of Modena and Reggio Emilia Modena Italy; ^8^ Department of Neurosciences Azienda Ospedaliero‐Universitaria Di Modena Modena Italy; ^9^ ALS Center, Department of Neurology, Azienda Ospedaliero Universitaria Maggiore Della Carità University of Piemonte Orientale Novara Italy

## Abstract

**Objective:**

The objective of this study was to evaluate the prognostic value of several muscle damage biomarkers.

**Methods:**

Data from Piemonte and Valle d'Aosta Amyotrophic Lateral Sclerosis (PARALS) were considered for this study. Survival was defined as the time from diagnosis to death, tracheostomy, or the censoring date. Blood levels of potassium, creatinine, creatine kinase, phosphorus, aspartate aminotransferase (AST) and alanine aminotransferase (ALT) diagnosis were evaluated as potential prognostic biomarkers. A Cox model was developed for each biomarker and adjusted for sex, onset age, onset site, and diagnostic delay. Significant findings from PARALS were evaluated in the Pooled Resource Open‐Access Amyotrophic Lateral Sclerosis Clinical Trials (PRO‐ACT) database. Additionally, a joint model was constructed to evaluate the prognostic role of phosphatemia slope over time using longitudinal data from PRO‐ACT.

**Results:**

A total of 1,444 and 1,023 patients were included in the PARALS and PRO‐ACT cohorts, respectively. Only creatinine (hazard ratio [HR] = 0.65, 95% confidence interval [CI] = 0.50–0.85) and phosphorus (HR = 1.14, 95% CI = 1.04–1.24) showed a significant association with survival in the PARALS cohort. These findings were further validated in the PRO‐ACT cohort (creatinine HR = 0.21, 95% CI = 0.13–0.35, *p* < 0.0001; phosphorus HR = 2.35, 95% CI = 1.13–4.88, *p* = 0.02). Longitudinal data from the PRO‐ACT database showed that an increase of 0.1 mmol/l per month in phosphate levels was also associated with a HR of 8.26 (95% CI = 1.07–96.6, *p* = 0.044).

**Interpretation:**

Creatininemia was confirmed as a prognostic marker in amyotrophic lateral sclerosis (ALS). Additionally, both phosphatemia levels at diagnosis and its rate of change over time were identified as a potential prognostic marker for ALS. As with other blood biomarkers, phosphate levels are cost‐effective and minimally invasive to measure, supporting their potential use in clinical trials. ANN NEUROL 2025;98:286–293

Amyotrophic lateral sclerosis (ALS) is a devastating disease, characterized by the relentless paralysis of voluntary muscles and ultimately leading to death, usually due to respiratory failure. Although the median survival time from symptoms onset to either death or tracheostomy is typically 2 to 5 years,^1^ this period varies widely, ranging from as short as a few months to more than 10 years.^2^ Understanding and accurately predicting this variability is essential for patients and clinicians, and plays a crucial role for optimizing the design of clinical trials.

The search for reliable prognostic factors remains ongoing.^3^ Some factors, such as age and site of onset, the phenotype at diagnosis, and certain genetic variants, are well‐established.^2^ However, one of the most reliable predictors is the extent of disease‐related damage at a given timepoint (usually the time of diagnosis).^2^, ^3^ Over time, this damage has been assessed through different clinical measures, including the total score of the revised ALS Functional Rating Scale (ALSFRS‐R),^4^ or more accurately, the slope of its decline from onset to diagnosis,^5^ as well as the King's College staging system,^6^ the Medical Research Council Scale,^7^ and pulmonary functional tests.^8^, ^9^ Although the decline in these measures may vary throughout the disease course,^10^, ^11^ it is generally accepted that lower scores at diagnosis typically indicate a poorer prognosis.^2^, ^3^

Nonetheless, the ALSFRS‐R have several limitations, making it inadequate for precisely capturing the ongoing damage to motor neurons. This is partly due to its discrete nature (despite its use as a continuous measure) and its multidimensionality, which can result in patients with the same total score exhibiting significantly different degree of impairment.^12^, ^13^ Consequently, significant efforts have been directed toward the identification of biomarkers that can reliably, sensitively, and objectively quantify the motor neuron damage and serve as prognostic or predictive factors.^14^

Given that motor neuron impairment in ALS leads to muscle wasting, any biomarker reflecting either acute muscle damage or chronic muscle loss could potentially serve as a prognostic biomarker in ALS.^15^

Accordingly, here, we utilized 2 large cohorts of patients with ALS—one population‐based and one multicentric, incorporating data from multiple clinical trials—to assess the prognostic value of several muscle damage biomarkers.

## Methods

Patients from the Piemonte and Valle d'Aosta ALS (PARALS) database register diagnosed between 2007 and 2019 were considered for the study. The register collects data on patients diagnosed with ALS who were residents of either Piemonte or Valle d'Aosta, Italy, at the time of diagnosis. The primary data sources are the 2 ALS tertiary centers located in Torino and Novara, with additional information periodically obtained from the administrative databases of major hospitals in both regions. Patients are included in the PARALS if they meet the criteria for definite, probable, probable laboratory‐supported, or possible ALS according to the revised El Escorial Diagnostic Criteria (EECr),^16^ at any stage of the disease.^17^ For this study, patients diagnosed with primary lateral sclerosis (PLS) were excluded.

At the 2 ALS tertiary centers, the diagnostic workup includes a hematological examination, with blood samples collected after overnight fasting. For patients diagnosed in general neurological clinics (n = 44), hematological data from the time of diagnosis were retrieved from the medical records.

For the purposes of this study, we considered the following hematological tests as potential markers of acute or chronic muscle damage: creatinine (mg/dl), creatine phosphokinase (IU/l), potassium (mmol/l), alanine aminotransferase (ALT; IU/l), aspartate aminotransferase (AST; IU/l), and phosphorus (mmol/l; hereinafter, interchangeably referred to as phosphorus or phosphate).^18^, ^19^ To improve interpretability, creatine phosphokinase levels were divided by 100 (to measure the hazard ratio [HR] for each 100 UI/l increase), whereas ALT and AST levels were divided by 10 (HR for each 10 UI/l increase). None of the patients included in the study had renal failure or were receiving riluzole at the time of blood testing.

Data from the Pooled Resource Open‐Access Amyotrophic Lateral Sclerosis Clinical Trials (PRO‐ACT) database were used to confirm our results. This open‐access database contains records from over 10,000 patients who participated in 23 phase II/III clinical trials and 1 observational study. For our analysis, we selected patients with available survival data and the specified hematological tests taken within 90 days of study entry. The data were obtained from the latest version of the PRO‐ACT database (August 1, 2022). To extract demographic, laboratory, and survival information, we merged the ALS History, Death Data, Demographics, ALSFRS, and Labs datasets using the “subject_id” as the key identifier. Detailed information on the data contained in PRO‐ACT, as well as its inclusion and exclusion criteria, is provided elsewhere.^20^

## Statistical Analysis

Survival from diagnosis was considered in both cohorts as it aligned with the timing of biomarker measurements, which were taken at the time of diagnosis, and because the diagnosis date is usually more precisely defined than the disease onset date. Accordingly, in the PARALS cohort, survival was defined as the time from diagnosis to death, tracheostomy, or the censoring date (set as December 31, 2023). Comparisons of medians (reported with interquartile ranges [IQRs]) and proportions were assessed using the Mann–Whitney test and Chi‐square test, respectively. Survival analyses were performed using the Kaplan–Meier method and compared with the log rank test.

After verifying the proportional hazards assumption with the Schoenfeld test, multiple Cox proportional hazards regression models were constructed for each blood marker of interest (considered as a continuous variable). These models were adjusted for sex, age at onset, diagnostic delay (defined as the time from symptom onset to diagnosis, expressed in months), and site of onset (categorized as bulbar or spinal). The HRs with 95% confidence intervals (95% CIs) were calculated. A *p* value of < 0.05 was considered statistically significant, with Bonferroni correction applied for multiple testing (*p* < 0.008). Given the potential correlation between blood phosphate levels and respiratory acidosis, we also calculated Spearman's Rho between phosphate levels and force vital capacity (FVC) measured at diagnosis.

Blood markers that were found to be statistically significant in the PARALS cohort were subsequently evaluated in the PRO‐ACT database. In the PRO‐ACT cohort, survival was defined as the time from diagnosis to either death or the last follow‐up date. The Cox model was adjusted for sex, age at onset, diagnostic delay (time from symptom onset to diagnosis, expressed in months), and site of onset (categorized as bulbar or spinal). Creatinine levels were converted from μmol/l to mg/dl in order to ensure consistency with the PARALS cohort.

After having assessed the prognostic role of baseline phosphorus levels, we leveraged the availability of longitudinal measurements in the PRO‐ACT database to evaluate whether the slope of phosphorus levels over time also was prognostic of ALS survival. To achieve this, we first used a linear mixed‐effects model with phosphorus levels as the dependent variable, adjusting for the same patient characteristics as the fixed effects. Time from trial entry was modeled as a random slope, whereas patient ID was included as a random intercept. Next, we constructed a Cox proportional hazards model incorporating the same independent predictors of ALS survival as previous Cox models. Finally, a joint model was fitted to simultaneously account for the longitudinal phosphorus trajectories and their association with survival. The model estimation was performed using a Markov Chain Monte Carlo (MCMC) approach with 12,000 iterations, a burn‐in period of 2,000 iterations, and a thinning interval. We explored models incorporating time‐dependent slopes (ie, phosphorus trajectories) with and without the effect of the current phosphorus value. Model selection was guided by the Deviance Information Criterion (DIC), the Watanabe‐Akaike Information Criterion (WAIC), and the Log Pseudo‐Marginal Likelihood (LPML). HRs with 95% CIs and corresponding *p* values from the selected model were reported.

The study was approved by the Ethical Committee of the Turin ALS Center (Comitato Etico Azienda Ospedaliero‐Universitaria Città della Salute e della Scienza, Torino, #0038876).

## Results

A total of 1,797 patients were diagnosed during the study period within the PARALS cohort. Blood test results were available for 1,444 (80.4%) patients (Table 1), with no relevant clinical or demographic differences between those included in the analysis and those who were not (Supplementary Table S1). Each biomarker was available for a different subset of patients (Supplementary Table S2) and there were no relevant differences between patients with available data and those without (Supplementary Tables S3 and S4; only comparisons for phosphorus and creatinine are reported). During the follow‐up period, 1,128 (78.1%) patients died, whereas 205 (14.2%) underwent tracheostomy (among these, only 11, 0.7%, were alive at the time of censoring), with a median survival time from diagnosis of 1.65 years (IQR = 0.79–3.09 years).

**TABLE 1:** Demographical and Clinical Characteristic of Patients Included in the PARALS and PROACT Cohorts

	PARALS Cohort (n = 1,444)	PROACT Creatinine Cohort (n = 1,023)	*p*	PROACT Phosphorus Cohort (n = 638)	*p*
Sex, M (%)	812 (56.2)	634 (62.0)	**0.005**	393 (61.6)	**0.025**
Onset age, yr, median (IQR)	68.7 (60.8–74.7)	58.0 (51.0–66.0)	**< 0.001**	58.0 (51.0–65.0)	**< 0.001**
Onset site, spinal (%)	958 (66.3)	802 (78.4)	**< 0.001**	495 (77.6)	**< 0.001**
Diagnostic delay, mo median (IQR)	9.6 (5.6–13.6)	9.1 (5.6–14.1)	0.321	8.8 (5.3–12.9)	**0.027**

Note: The figures in bolds refer to statistically significant results.

IQR = interquartile range.

**FIGURE 1:**
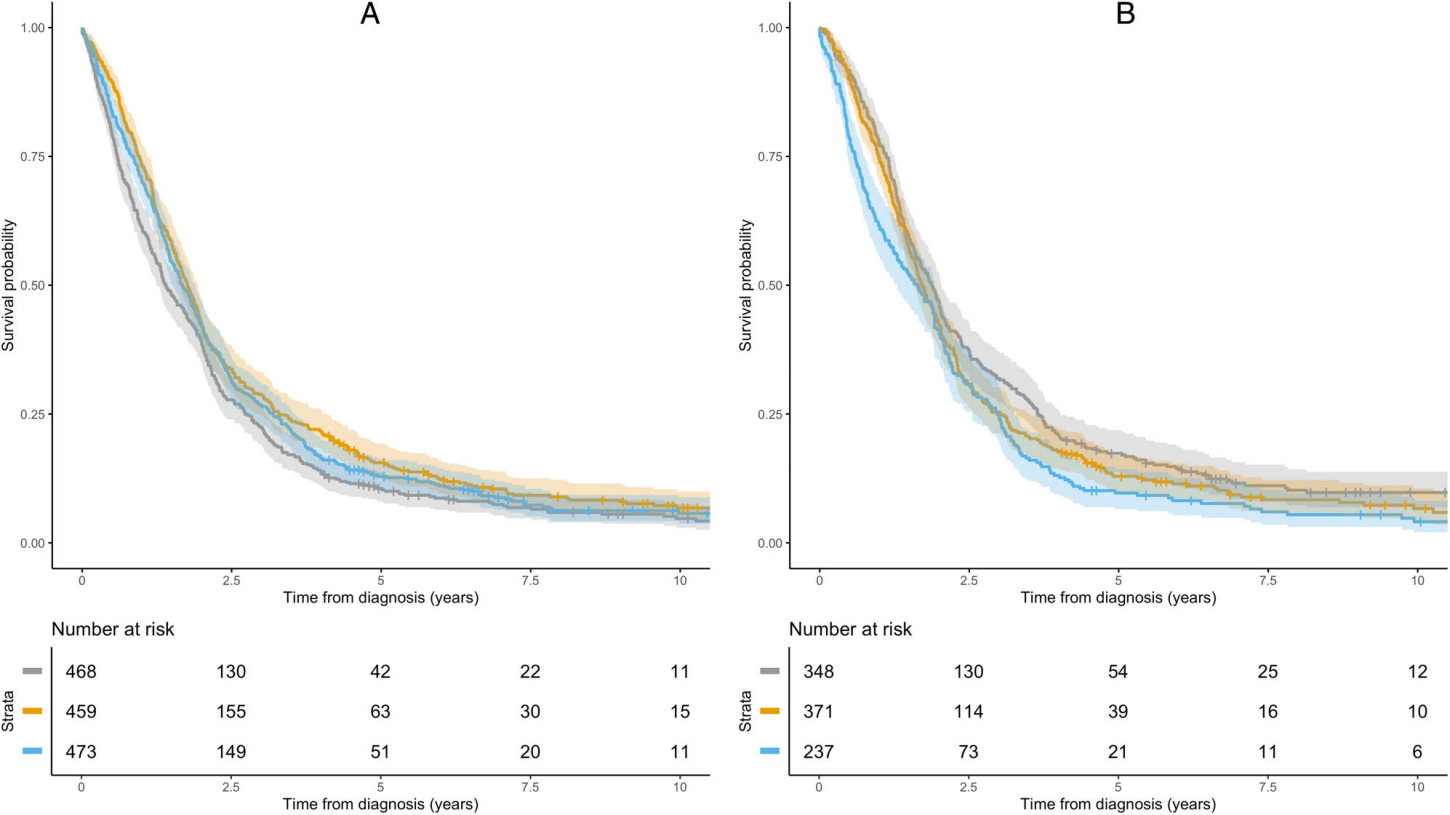
Kaplan–Meier curves and risk tables stratified according to creatinine (A) and phosphorus (B) tertiles in the PARALS cohort. Grey = lower tertiles; yellow = medium tertiles; blue = higher tertiles. PARALS = Piemonte and Valle d'Aosta Amyotrophic Lateral Sclerosis. [Color figure can be viewed at www.annalsofneurology.org]

In the PARALS cohort, only creatinine and phosphorus were significantly correlated with survival from the time of diagnosis, after accounting for multiple testing (Table 2). The median creatinine level was 0.74 mg/dl (IQR = 0.61–0.88 mg/dl). Median survival increased between the first and second tertiles of creatinine levels (from 1.4 to 1.76 years), before slightly decreasing in the third tertile (1.64 years). When analyzed as a continuous variable, creatinine was associated with survival with an adjusted HR of 0.65 (95% CI = 0.50–0.85, *p* = 0.001). Given that men generally have higher muscle mass than women, we also conducted a sex‐specific analysis for creatininemia. The results were consistent with the overall cohort, showing an HR of 0.69 (95% CI = 0.51–0.93, *p* = 0.017) for men, and an HR of 0.59 (95% CI = 0.36–0.97, *p* = 0.037) for women. Conversely, phosphorus levels were inversely correlated with survival, which declined steadily across its tertiles (from 1.86 to 1.72 to 1.64 years), with an adjusted HR of 1.14 (95% CI = 1.05–1.24, *p* = 0.001; see Table 2 and the Fig 1). Phosphorus levels were generally within the normal range, with a median value of 1.1 mmol/l (IQR = 1.00–1.20), except for 1 patient who had elevated levels (4.8 mg/dl). A total of 860 (59.5%) patients had both phosphate levels and FVC measurement at the time of diagnosis, but no correlation was observed between these 2 measures (rho = − 0.04, *p* = 0.19).

**TABLE 2:** Cox Univariate and Multivariable Model in the PARALS Cohort

Blood Biomarker	Univariate Analysis		Multivariable Analysis	
	HR (95% CI)	*p*	Adjusted HR (95% CI)	*p*
Creatine kinase	0.96 (0.93–0.99)	0.010	0.98 (0.95–1.00)	0.089
Creatinine	0.98 (0.8–1.18)	0.796	0.65 (0.50–0.85)	**0.001**
Phosphorus	1.15 (1.06–1.24)	< 0.001	1.14 (1.05–1.24)	**0.001**
Potassium	1.01 (0.99–1.03)	0.241	1.00 (0.99–1.02)	0.611
AST	1.04 (0.99–1.09)	0.094	1.04 (0.99–1.09)	0.083
ALT	1.00 (0.98–1.03)	0.834	1.01 (0.99–1.04)	0.309

Note: The figures in bold refer to statistically significant results.

ALT = alanine aminotransferase; AST = aspartate aminotransferase; 95% CI = 95% confidence interval; HR = hazard ratio.

In the PRO‐ACT database, 1,023 patients had data on survival, model covariates, and creatinine, whereas 638 patients had data on survival, covariates, and phosphorus. As expected, the clinical and demographic characteristics of the PRO‐ACT cohort differed from those of the PARALS cohort, with PRO‐ACT patients more often being men, younger at disease onset, and more likely to present with spinal onset (see Table 1). In the creatinine cohort, 499 patients (49.8%) died, with a median survival of 0.67 years (IQR = 0.40–0.92 years). In the phosphorus cohort, 367 patients (57.5%) died, with a median survival of 0.70 years (IQR = 0.39–0.96 years). The multivariable analysis confirmed the prognostic value of both creatinine and phosphorus levels with an adjusted HR of 0.21 (95% CI = 0.13–0.35, *p* < 0.0001) and 2.35 (95% CI = 1.13–4.88, *p* = 0.02), respectively. Accordingly, survival increased steadily across creatinine tertiles (from 0.84 to 1.02 to 1.03 years), whereas it decreased, albeit not linearly, across phosphorus tertiles (from 0.97 to 0.99 to 0.92 years).

In the PRO‐ACT database, 578 patients had at least 2 longitudinal phosphorus measurements, with a median of 5.5 evaluations over a median follow‐up of 182 days from clinical entry. After fitting the joint models and evaluating the information criteria, we selected the model that included only the time‐dependent slope of phosphate levels (Supplementary Table S5). According to this model, an increase of 0.1 mmol/l per month in phosphate levels was associated with an HR of 8.26 (95% CI = 1.07–96.6, *p* = 0.044).

## Discussion

Using a large population‐based cohort of patients with ALS, we assessed the prognostic value of various blood markers related to muscle damage and loss. Among the 6 biomarkers tested at the time of diagnosis, only creatinine (HR = 0.65) and phosphate levels (HR = 1.14) were significantly associated to survival. Using data from the PRO‐ACT database, we confirmed the positive relationship of creatinine levels and the negative relationship of phosphorus with survival in patients enrolled in multiple clinical trials. Furthermore, based on longitudinal measurements from the PRO‐ACT database during trial follow‐up, we found that the rate of increase in phosphorus levels over time (ie, the slope) was also negatively associated with ALS prognosis (HR = 8.26 per 0.1 mmol/L increase per month), as previously found for the slope of creatinine levels.^21^

There is an unmet need for reliable prognostic biomarkers in ALS, providing patients with more accurate predictions and aiding physicians in planning treatment and care more effectively. More importantly, objective biomarkers could significantly improve clinical trial design by enabling patient stratification based on disease progression rate, which would enhance statistical power and reduce study costs.^21^ Additionally, biomarkers could serve as objective and easy‐to‐measure end points, as opposed to fatal and functional end points commonly used in current studies.^21^, ^22^ In some cases, biomarkers may also cluster patients based on distinct disease mechanisms, potentially identifying therapeutic targets.^14^ Finally, in asymptomatic patients carrying pathogenetic ALS variants, biomarkers could predict the symptoms onset, aiding the timely application of targeted therapies.^14^

Whereas neurofilaments light chain (NfL) currently represent the most prominent example of a biomarker being implemented as an end point in ALS clinical trials,^23^ many other wet, imaging, and neurophysiology biomarkers are under active investigation.^14^ In a field with so many needs, it is unlikely that any single biomarker will address all of them.

Markers of muscle damage could meet some of these goals.^15^ Although not ideal for discovering new pathogenetic mechanisms, these markers could be valuable for objectively tracking disease progression in both symptomatic and presymptomatic patients. They could also help stratify patients for clinical trials and serve as a reliable primary or secondary outcome.

A variety of markers of muscle involvement has been investigated as potential prognostic biomarkers for ALS, including potassium,^24^, ^25^ creatine kinase,^15^, ^24^, ^26^ lactate dehydrogenase,^24^ myoglobin,^15^ troponin T,^15^, ^27^ AST, ALT,^24^ and creatinine.^21^, ^25^, ^28^, ^29^, ^30^

In this context, the prognostic role of creatinine we observed in this study was not unexpected. Creatinine is a byproduct of creatine phosphate breakdown in muscles and is excreted unchanged by the kidneys. Therefore, whereas it is often used as an indicator of renal function, it also reflects overall muscle mass. The direct relationship between creatinine levels at diagnosis and survival in patients with ALS has already been demonstrated in a smaller subgroup of the PARALS cohort. In patients diagnosed between 2007 and 2011, creatinine levels below 0.82 mg/dl for men and 0.65 mg/dl for women were associated with a reduced survival (HR = 1.49 and HR = 1.47, respectively).^28^ In the PRO‐ACT cohort, both baseline creatinine levels^20^, ^29^ and their longitudinal decline were associated with mortality, as well as more rapid decline of ALSFRS‐R and muscle strength.^21^ These findings have been replicated across several real‐world cohorts,^25^, ^31^, ^32^ also reporting a significant correlation between creatinine levels and lower motor neuron damage.^33^ Two systematic reviews further confirmed that baseline creatinine decline is inversely correlated with functional score deterioration^30^ and directly correlated with survival.^34^

In contrast, phosphate is not typically included in the routine blood panels of patients with ALS in real‐world settings, and therefore has not been considered in prior analyses. It has only been featured in a few studies utilizing data from the PRO‐ACT database, where machine learning algorithms were applied to predict ALS survival or intermediate milestones. In these studies, phosphorus was identified as a significant contributor to accurate predictions of ALS progression.^35^, ^36^, ^37^

Phosphorus, absorbed primarily in the small intestine, is mostly stored in bones, with about 10% located in soft tissues like the liver and skeletal muscle, and about 5% circulating in the blood as phosphate. Beyond its structural role as a component of hydroxyapatite in bones, phosphorus is a building block of phospholipids, a component of nucleotides in DNA and RNA and crucial for cellular energy metabolism, as it is contained in the adenosine triphosphate (ATP) molecule. Phosphorus metabolism is regulated by several factors, including parathyroid hormone (PTH), which increases its release from bones and promotes renal excretion to prevent calcium phosphate crystal formation; fibroblast growth factor 23 (FGF23), which also enhances renal excretion; vitamin D, which boosts intestinal absorption; and insulin, which drives phosphate into cells. As a result, there are multiple causes of hyperphosphatemia, including excessive intake (often iatrogenic or dietary in the context of chronic kidney disease), decreased renal excretion (as seen in renal failure), or abnormal transcellular shifts, such as in rhabdomyolysis, tumor lysis, or hypoinsulinemia.^38^, ^39^ Chronic respiratory acidosis may also elevate phosphate levels due increased cellular efflux.^38^

The aim of this study was to identify prognostic factors, rather than establish causal relationship with ALS survival. However, among the factors that can elevate phosphate levels, muscle damage represents a plausible confounder explaining the observed association between phosphate levels and survival. Although we cannot rule out the potential role of respiratory acidosis, which can occur in patients with ALS, the lack of correlation between phosphate levels and respiratory function tests makes this possibility less likely.

Although muscle damage is a likely explanation, we acknowledge the absence of data on insulin levels. Moreover, due to the study's observational design, we cannot fully exclude that higher levels of phosphate themselves might reduce survival. In fact, in patients with chronic renal failure, hyperphosphatemia—or even increased levels within the normal range—has been associated with increased mortality, likely due to vascular calcification and a higher risk of cardiovascular complications.^40^ However, it must be noted that cardiovascular events are known to be infrequent in the ALS population.^41^

The population‐based design of the PARALS cohort and the use of another independent large cohort are significant strengths of this study, but some limits must be acknowledged. First, the absence of longitudinal data in the PARALS cohort prevented us from confirming the prognostic role of the phosphate level slopes over time and from obtaining a more precise estimate, given the wide CIs in the PRO‐ACT database. This also limits our ability to determine if interventions, such as riluzole treatment, multidisciplinary care, or early treatment with noninvasive ventilation might influence phosphate levels. Additionally, although the study was population‐based, phosphate measurements were only available for a subset of patients. Despite this, the lack of significant differences between included and excluded patients based on available characteristics suggests that selection bias is unlikely. Last, the PRO‐ACT cohort showed notably different HRs for both creatinine and phosphate. This discrepancy likely reflects the well‐known selection bias in clinical trials or confounders differing across the 2 cohorts and that were not included in the models.

In conclusion, our findings confirm that creatinine levels at diagnosis may serve as a prognostic marker in ALS. More importantly, we report for the first time that phosphate levels at diagnosis and its rate of change over time may also act as a prognostic marker, possibly due to its association with muscle damage. However, further studies are needed to confirm this explanation. Like other blood biomarkers, phosphate levels offer the advantages of being cost‐effective and minimally invasive, positioning it as a promising candidate for use in clinical trials.

## Authors Contributions

R.V. contributed to the conception and design of the study, to the acquisition and analysis of data, and to drafting of the text and preparing the figures. E.K. contributed to the design of the study design, to the analysis of data and to drafting the text. A.Can., U.M., M.G., F.P., S.C., E.M., F.D.P., F.D.Mat., F.V., J.M., C.S., I.M., F.D.Mar., L.M., C.M., A.Cal., and A.Chi. contributed to acquisition of data and to drafting of the text.

## Potential Conflicts of Interest

Nothing to report.

## Supporting information


**Supplementary Table S1.** Demographical and clinical characteristics of patients from the PARALS cohort during the study period, overall and based on their inclusion in the study.
**Supplementary Table S2.** Data availability for each biomarker assessed in the PARALS cohort (n = 1,444).
**Supplementary Table S3.** Demographical and clinical characteristics of patients from the PARALS cohort based on the availability of the phosphorus blood levels at the time of diagnosis.
**Supplementary Table S4.** Demographical and clinical characteristics of patients from the PARALS cohort based on the availability of the creatinine blood levels at the time of diagnosis.
**Supplementary Table S5.** Comparison of joint models assessing the prognostic role of phosphorus trajectories in the PRO‐ACT database.

## Data Availability

Data are available to interested researchers upon motivated and reasonable request.
