# Supplementary material for: Phosphatemia is an Independent Prognostic Factor in Amyotrophic Lateral Sclerosis
Source: Ann Neurol. 2025 Apr 26;98(2):286–93. doi: 10.1002/ana.27252 (PMC12278033; doi:10.1002/ana.27252)
Supplement: Supplementary file 1 — Supplementary Table S1. Demographical and clinical characteristics of patients from the PARALS cohort during the study period, overall and based on their inclusion in the study. Supplementary Table S2. Data availability for each biomarker assessed in the PARALS cohort (n = 1,444). Supplementary Table S3. Demographical and clinical characteristics of patients from the PARALS cohort based on the availability of the phosphorus blood levels at the time of diagnosis. Supplementary Table S4. Demographical and clinical characteristics of patients from the PARALS cohort based on the availability of the creatinine blood levels at the time of diagnosis. Supplementary Table S5. Comparison of joint models assessing the prognostic role of phosphorus trajectories in the PRO‐ACT database. [file ANA-98-286-s001.doc]

**Supplementary table 1.** Demographical and clinical characteristics of patients from the PARALS cohort during the study period, overall and based on their inclusion in the study.

|  | **Overall (n =1797)** | **Included (n=1444)** | **Not included (n=353)** | **p-value** |
| --- | --- | --- | --- | --- |
| Sex, M (%) | 992 (55.2) | 812 (56.2) | 180 (51.0) | 0.086 |
| Onset age, years [median (IQR)] | 68.9 (60.8-74.9) | 68.7 (60.8-74.7) | 69.3 (60.8-76.5) | 0.183 |
| Onset site, bulbar (%) | 612 (34.1) | 486 (33.7) | 126 (35.7) | 0.508 |
| Diagnostic delay, months [median (IQR)] | 9.6 (5.6-13.7) | 9.6 (5.6-13.6) | 10.6 (6.5-17.7) | **0.007** |

**Supplementary table 2.** Data availability for each biomarker assessed in the PARALS cohort (n=1444).

| **Blood biomarker** | **Patients**  **with missing data (%)** |
| --- | --- |
| Creatine kinase | 160 (11.1) |
| Creatinine | 44 (3.0) |
| Phosphorus | 488 (33.8) |
| Potassium | 67 (4.6) |
| Aspartate aminotransferase (AST) | 571 (39.5) |
| Alanine aminotransferase (ALT) | 541 (37.4) |

**Supplementary table 3.** Demographical and clinical characteristics of patients from the PARALS cohort based on the availability of the phosphorus blood levels at the time of diagnosis.

|  | **Phosphorus**  **not available (n=488)** | **Phosphorus available**  **(n=956)** | **p-value** |
| --- | --- | --- | --- |
| Sex, M (%) | 258 (52.9) | 554 (57.9) | 0.074 |
| Onset age, years [median (IQR)] | 69.7 (61.6-75.2) | 68.2 (60.4-74.5) | **0.049** |
| Onset site, bulbar (%) | 158 (32.4) | 328 (34.3) | 0.499 |
| Diagnostic delay, months [median (IQR)] | 9.6 (5.6-13.7) | 8.6 (5.6-13.6) | 0.827 |

**Supplementary table 4.** Demographical and clinical characteristics of patients from the PARALS cohort based on the availability of the creatinine blood levels at the time of diagnosis.

|  | **Creatinine**  **not available (n=44)** | **Creatinine available**  **(n=1400)** | **p-value** |
| --- | --- | --- | --- |
| Sex, M (%) | 24 (54.5) | 788 (56.3) | 0.074 |
| Onset age, years [median (IQR)] | 67.4 (61.3-74.6) | 68.8 (60.8-74.7) | **0.049** |
| Onset site, bulbar (%) | 12 (27.3) | 474 (33.9) | 0.499 |
| Diagnostic delay, months [median (IQR)] | 10.6 (7.5-15.4) | 9.6 (5.6-13.6) | 0.827 |

**Supplementary table 5**. Comparison of joint models assessing the prognostic role of phosphorus trajectories in the PROACT database.

| **Model** | **DIC** | **WAIC** | **LPML** |
| --- | --- | --- | --- |
| Slope | 12525.96 | 12513.02 | -6259.337 |
| Current value | 12524.50 | 12514.62 | -6264.159 |
| Current value and slope | 12514.11 | 12522.13 | -6283.415 |

DIC = Deviance Information Criterion; WAIC = Watanabe-Akaike Information Criterion; LPML = Log Pseudo-Marginal Likelihood.

The criteria are calculated based on the marginal log-likelihood.
